# Optimisation of Convolution-Based Image Lightness Processing

**DOI:** 10.3390/jimaging10080204

**Published:** 2024-08-22

**Authors:** D. Andrew Rowlands, Graham D. Finlayson

**Affiliations:** Colour & Imaging Lab, School of Computing Sciences, University of East Anglia, Norwich NR4 7TJ, UK; g.finlayson@uea.ac.uk

**Keywords:** lightness, retinex, convolution filter, least squares optimisation

## Abstract

In the convolutional retinex approach to image lightness processing, an image is filtered by a centre/surround operator that is designed to mitigate the effects of shading (illumination gradients), which in turn compresses the dynamic range. Typically, the parameters that define the shape and extent of the filter are tuned to provide visually pleasing results, and a mapping function such as a logarithm is included for further image enhancement. In contrast, a statistical approach to convolutional retinex has recently been introduced, which is based upon known or estimated autocorrelation statistics of the image albedo and shading components. By introducing models for the autocorrelation matrices and solving a linear regression, the optimal filter is obtained in closed form. Unlike existing methods, the aim is simply to objectively mitigate shading, and so image enhancement components such as a logarithmic mapping function are not included. Here, the full mathematical details of the method are provided, along with implementation details. Significantly, it is shown that the shapes of the autocorrelation matrices directly impact the shape of the optimal filter. To investigate the performance of the method, we address the problem of shading removal from text documents. Further experiments on a challenging image dataset validate the method.

## 1. Introduction

It is well known that the retinex theory [1,2] of colour vision pioneered by Land postulates that the human visual system (HVS) has evolved to discount the illuminant. One consequence is that lightness, the psychophysical interpretation of luminance measured on a relative scale from dark to light, is thought to be more closely correlated with the relative reflectance of a scene object rather than its luminance [1].

The original retinex algorithms [1,2,3,4] were path-based computations; however, in 1986, Land proposed an alternative approach that could be interpreted as the convolution of an image with a centre/surround filter [5]. The idea was to remove shading (illumination gradients) by dividing the scene flux at each small area of interest by a weighted average of the flux from an extended surround that assumed a $1/r^{2}$ functional form. Convolutional retinex was developed further and applied to digital images by Jobson et al. using a Gaussian surround [6]. They also developed a multiscale retinex (MSR) that uses a sum of Gaussians of different spatial extents [7] and MSR with colour restoration (MSRCR) [8]. If we consider the single-scale retinex [6], the output can generally be modeled as

(1) $$I\left(x,y\right)=g\left(\frac{c(x,y)}{s(x,y)\;*\;c(x,y)}\right),$$

where *c* is the input image, *s* is the surround component of the centre/surround filter, “∗” denotes convolution, and *g* is a global mapping function that scales the output to the desired range [9].

Land [5], when performing his analogue experiments, took the global mapping function, *g*, to be the logarithmic function in order to approximate the nonlinear relationship between relative reflectance and lightness as perceived by the HVS [10]. Significantly, Jobson et al. [6,7] also took *g* to be the logarithmic function. However, it has since been argued that such a nonlinear mapping is not appropriate when dealing with retinex output displayed on electronic devices [9,11], and some authors proposed a simple linear stretch to the output range instead [11,12,13]. Indeed, the convolutional retinex algorithm of Jobson et al. [6,7] renders image lightness, particularly in darker areas of the image, in a manner that goes beyond the original premise of retinex, which was simply to mitigate gradients in the illumination, i.e., shading. Instead, Jobson et al.’s algorithm can be regarded as a local tone-mapping operator (TMO) for image enhancement that is tuned to provide visually pleasing images [8,14,15].

Consequently, algorithms based on MSR [7,8,9,11,16,17] and variations/extensions have been applied in diverse areas of image enhancement such as multi-sensor fusion [18], HDR tone mapping, e.g., [19], medical imaging, e.g., [20], night-time image enhancement, e.g., [21,22], underwater image enhancement, e.g., [23], image dehazing, e.g., [24], and aerial image enhancement, e.g., [25]. Moreover, many other types of image enhancement algorithms that take inspiration from the HVS have also been classed as retinex-based methods. For example, see Refs. [26,27,28] for some recent surveys.

However, recall that the original premise of retinex in the context of lightness perception was that the HVS discounts shading (gradients in the illumination), which means that lightness is thought to be more closely correlated with the relative reflectance of a scene object rather than its luminance. This recently led us to introduce a statistical approach to convolutional retinex that, in contrast to the above image enhancement methods, solely aims to mitigate shading from images in an objective manner [29]. The method produces convolution filters that are optimal, in a least squares sense, for removing shading from specific categories of scenes or image datasets. The key quantities required are estimates of the autocorrelation matrices for the image albedo (reflectance) and shading components. Then, via a model-based approach, the optimal filter can be obtained in closed form. Consequently, situations where the autocorrelation statistics can be more accurately estimated, for example, where the shadings have a known functional form, will lead to a more effective optimal filter. The method is an analytic reformulation of the earlier numerical approach of Hurlbert and Poggio, who developed a novel least squares formulation of retinex back in 1988 [30,31].

Since our goal is to design optimal filters for removing shading rather than to enhance images for viewing preference, we take the global mapping function *g* of Equation (1) to be linear, or, more specifically, to be a division by the 99.7th quantile as described later in Section 3.6. We actually carry out the filtering in the logarithmic domain in order to facilitate the separation of illumination and albedo by transforming their product into a sum, which enables us to apply our linear least squares optimisation; however, we then exponentiate the result.

**Figure 1:**
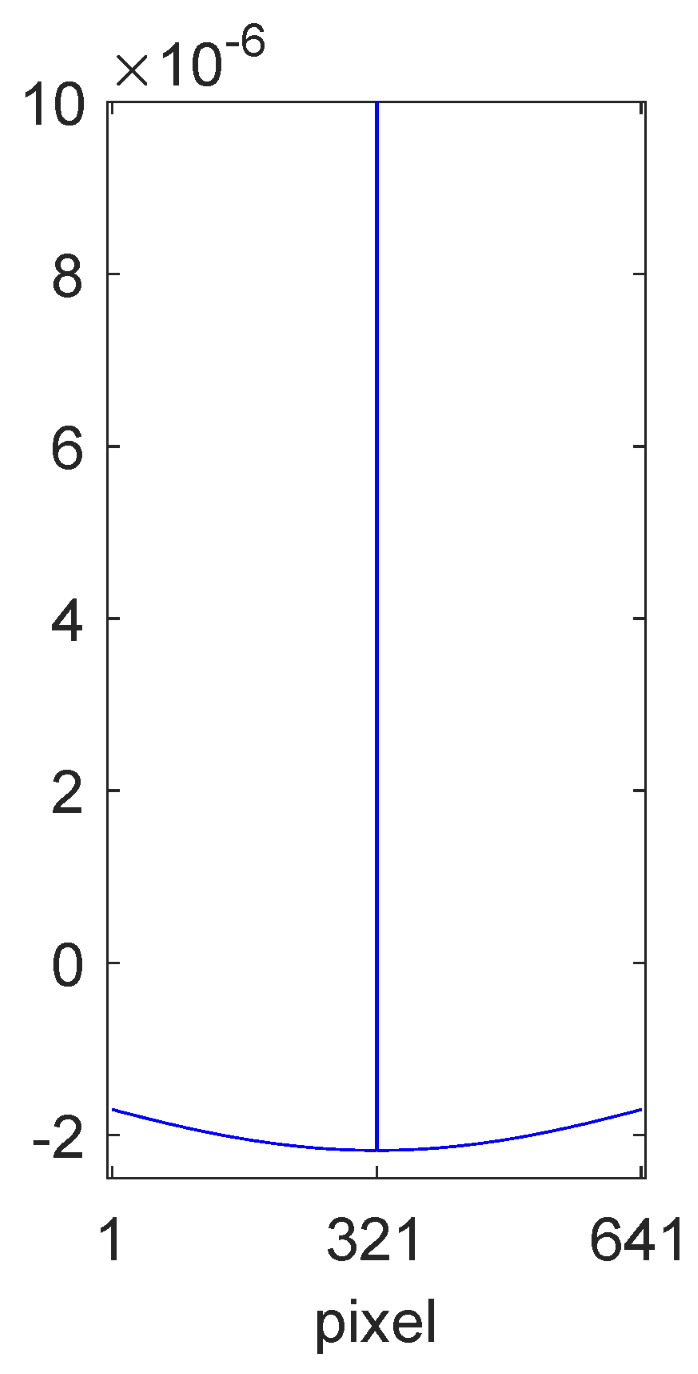
Cross section of our optimised convolution filter, $f_{2d}$, for the TM-DIED image dataset. The filter centre extends almost to unity but has been cropped close to the origin for clarity.

**Figure 2:**
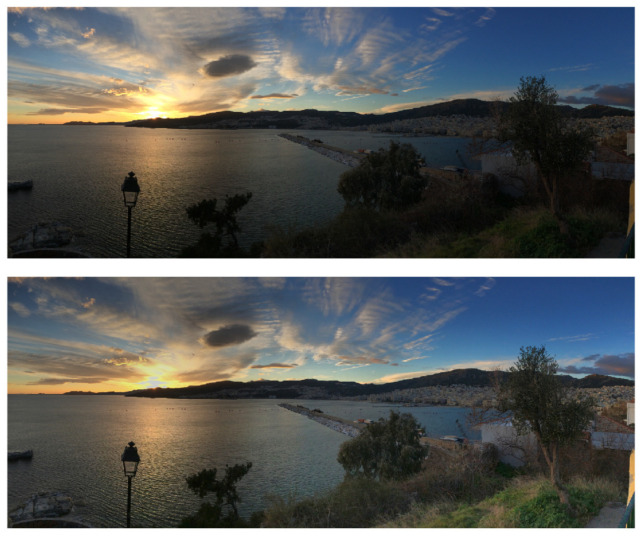
(**upper**) Example image from the TM-DIED dataset, which contains natural shading. (**lower**) Output image after convolving the upper image with the optimised convolution filter illustrated in Figure 1.

Figure 1 shows a cross-section of a centre/surround convolution filter (cropped near to the origin for clarity) computed using our method that was optimised for the TM-DIED image dataset [32]. This filter is derived later in this paper. An illustration of the type of output result to be expected is shown in Figure 2. The upper image is an example image from the TM-DIED dataset that contains natural shading due to the position of the setting sun. After convolving the logarithm of this image with the filter of Figure 1 and exponentiating the result, we arrive at the lower image of Figure 2, where it is clear that the shading has largely been removed. Note that in order to preserve chromaticity, we only filter the luminance channel [11,33].

Since the aim here is simply to objectively mitigate shading, it is important to notice that the filtered image result given in the lower image of Figure 2, along with the filtered image results given later in the final figure of this paper, are only subtly different from the corresponding input images. The results are not directly comparable with those of conventional convolutional retinex approaches, such as MSR, since the aim is different. As mentioned earlier, the parameters of the MSR algorithm are adjusted for subjective viewing preference, and a logarithmic rather than linear mapping function is applied to the convolved output. Furthermore, our filtered results will be very different from CNN-based image enhancement methods [34], such as LLCNN [35] and MBLLEN [36], where the aim is to enhance many aspects of image appearance.

The main contributions of this paper can be summarised as follows:

- We introduce a linear optimisation approach to convolutional retinex that mitigates shading (illumination gradients) from images. As described below, the theory is an analytic reformulation and extension of an earlier 1988 paper by Hurlbert and Poggio [30].
- The optimal linear filter adapts to known or estimated autocorrelation statistics of the albedo and illumination components of a given image training dataset. Consequently, the filter can be optimised for particular image datasets or scene categories. As discussed later in the paper, more accurate estimates of the autocorrelation matrices, for example situations where the illumination gradients have a known functional form, will lead to a better optimal filter.
- Since the filter can be obtained in closed form, the method is computationally very simple.
- Since our method is a simple linear approach where the aim is only to mitigate shading, the results will not be directly comparable to those of subjective image enhancement methods, including the single- or multiscale versions of convolutional retinex [6,7].
- Our method could be incorporated into more sophisticated (and computationally expensive) methods that utilise a linear step as part of their image enhancement processing or could be used as a preprocessing stage for training CNNs [34].

The next section of this paper begins with a brief summary of the original least squares optimisation approach to retinex taken by Hurlbert and Poggio [30]. Subsequently, full mathematical details of our analytic reformulation are provided, along with full implementation details, which were not presented in our earlier publication [29]. In Section 4, an optimal filter for removing shading applied to text documents is calculated. Significantly, this application provides an error analysis for the method since the original shading-free PDF pages can act as the ground truth. We also show how to determine an optimal filter for the TM-DIED dataset [32], which was designed to contain images taken in challenging lighting conditions.

## 2. Hurlbert and Poggio’s Method

We begin with a brief summary of the approach taken by Hurlbert and Poggio [30]. Let the colour signals (linear pixel values at image locations) be defined as

(2) $$c^{\prime }\left(x,y\right)=r^{\prime }\left(x,y\right)e^{\prime }\left(x,y\right),$$

where $r^{\prime }$ and $e^{\prime }$ are the image albedo and shading components, respectively, and x, y denote the pixel locations.

**Figure 3:**
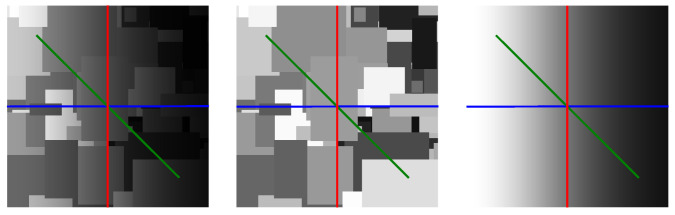
The example colour signal (**left**) is the product of the albedo image (**centre**) and shading image (**right**). The coloured lines are example corresponding scan lines or training vectors.

Now, suppose we have a large set of colour signals, randomly generated albedo images and randomly generated shading images. As illustrated in Figure 3, each colour signal must be the product of an albedo and shading image according to Equation (2). In the Hurlbert and Poggio method, a large number of training examples are taken in the form of image scan lines, i.e., one-dimensional (1d) training vectors of length *p* pixels extracted from the set of images. Three such example sets of corresponding scan lines are illustrated in Figure 3. (In order to preserve symmetry, flipped versions of all training vectors should be included in the training set). The training vectors can be arranged as rows of a set of matrices as follows:

(3) $$C^{\prime }=\left[\begin{array}{ccc} c_{11}^{\prime } & \cdots & c_{1p}^{\prime }\\ c_{21}^{\prime } & \cdots & c_{2p}^{\prime }\\ \; & \vdots & \\ c_{N1}^{\prime } & \cdots & c_{Np}^{\prime } \end{array}\right],\;R^{\prime }=\left[\begin{array}{ccc} r_{11}^{\prime } & \cdots & r_{1p}^{\prime }\\ r_{21}^{\prime } & \cdots & r_{2p}^{\prime }\\ \; & \vdots & \\ r_{N1}^{\prime } & \cdots & r_{Np}^{\prime } \end{array}\right],\;E^{\prime }=\left[\begin{array}{ccc} e_{11}^{\prime } & \cdots & e_{1p}^{\prime }\\ e_{21}^{\prime } & \cdots & e_{2p}^{\prime }\\ \; & \vdots & \\ e_{N1}^{\prime } & \cdots & e_{Np}^{\prime } \end{array}\right].$$

where *N* is the number of training vectors. Consequently,

(4) $$C^{\prime }=R^{\prime }⊙E^{\prime },$$

where ⊙ denotes the element-wise “Hadamard” product. However, by defining c(x,y) = $\log c^{\prime }\left(x,y\right)$, r(x,y) = $\log r^{\prime }\left(x,y\right)$, and e(x,y) = $\log e^{\prime }\left(x,y\right)$, Equation (2) can be converted to the following sum:

(5) c(x,y)=r(x,y)+e(x,y),

and so Equation (4) becomes

(6) C=R+E.

Now, let us introduce a p×p matrix operator *L* that relates the colour signal and albedo matrices,

(7) CL≈R.

By over-constraining the system so that N≫p, the optimum least squares solution is given by

(8) $$L={\left(C^{⊤}\;C\right)}^{\;-1}\;C^{⊤}\;R,$$

where ⊤ denotes the transpose operator and ${\left(C^{⊤}\;C\right)}^{\;-1}\;C^{⊤}$ is the Moore–Penrose pseudoinverse. When applied to any colour signal scan line c(x), the solved-for matrix operator *L* will *best* (in the least squares sense) recover the corresponding albedo scan line r(x).

In digital imaging, it is more convenient to use a convolution filter rather than a matrix operator. A 1d filter *f* can be extracted from *L* by simply taking the central column, in which case

(9) c ∗ f≈r,

where “∗” denotes convolution. (Note that in the case of a circularly shift-invariant system, *L* would be a circulant matrix, and so any column of *L* would be identical to the previous column but would be circularly shifted down by one pixel).

**Figure 4:**
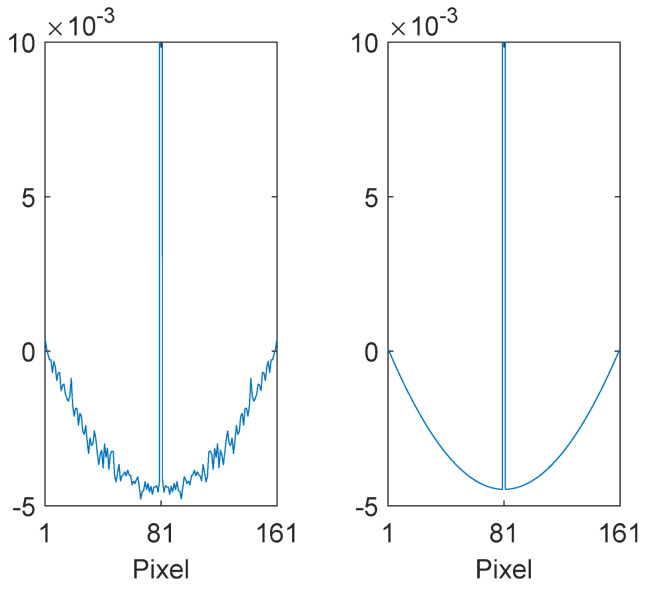
(**left**) Example one-dimensional filter of length *p* = 161 pixels obtained using Hurlbert and Poggio’s numerical method [30] with 1,000,000 pairs of training vectors. The illustration has been cropped close to the horizontal axis for clarity. (**right**) The corresponding filter obtained using our analytic reformulation.

The LHS of Figure 4 shows an example of a filter *f* obtained using the above method. The albedo images were taken to be random Mondrian images, and the shading images were a 50:50 mix of linear ramps and slowly varying sinusoids in the range [0.1351, 1], with a random wavelength and phase and with the minimum wavelength being four times the length of the training vectors. The optimum filter turns out to be a centre/surround filter, with a single pixel centre that extends almost to unity, and a very shallow negative surround. That is, in the logarithmic domain, we, at each pixel, remove the shading by subtracting a weighted average calculated over neighbouring pixels.

Evidently, the main drawback of the method is that the filter surround is very noisy. This is due to the relatively small number of training pairs (1,000,000 in this case) that can be utilised in practice. Noisy filters are unfeasible from a biological perspective and could also lead to artefacts when applied to real images. In contrast, the RHS of Figure 4 shows the smooth filter obtained using our analytic reformulation of Hurlbert and Poggio’s method that will be the main subject of the next section.

Although not suggested by Hurlbert and Poggio, *f* can be straightforwardly converted into a symmetric two-dimensional (2d) filter $f_{2d}$ simply by replicating the surround radially, interpolating as necessary [29]. Naturally, the surround subsequently needs to be normalised so that its sum equals that of the 1d surround. The symmetry of the training vectors will automatically be built into the filter. This can be applied in the Fourier domain to directly estimate complete two-dimensional albedo images as follows:

(10) $$F^{-1}\left(F\left(C\right)\;F\left(f_{2d}\right)\right)\approx R,$$

where *F* denotes the Fourier transform.

## 3. Derivation of an Optimal Lightness Convolution Filter in Closed Form

In this section, full mathematical details of our method are presented. The key steps are organised as follows:

- Given a training set of albedo vectors and shading vectors, an expression for a colour signal matrix that contains all possible combinations of these vectors is derived.
- Significantly, an analytic decomposition of the least squares solution is performed, which shows that the optimisation depends primarily upon $R^{⊤}\;R$ and $E^{⊤}\;E$, which are the autocorrelation matrices for the albedos and shadings, respectively.
- By introducing models for the albedo and shading training vectors, closed-form expressions for $R^{⊤}\;R$ and $E^{⊤}\;E$ are obtained by integrating over *all* possible training vectors.
- The algorithm and implementation details are discussed.

### 3.1. The Set of All Colour Signals

Let us proceed by constructing training sets of *n* albedo vectors {r(x)} and *m* shading vectors {e(x)}, all of which have length *p* pixels. As before, these are the logarithms of $\left\{r^{\prime }\left(x\right)\right\}$ and $\left\{e^{\prime }\left(x\right)\right\}$. The functional form for these vectors will be discussed later in Section 3.4 and Section 3.5. The vectors can be arranged as rows of the n×p matrix *R* and the m×p matrix *E* as follows:

(11) $$R=\left[\begin{array}{ccc} r_{11} & \cdots & r_{1p}\\ r_{21} & \cdots & r_{2p}\\ \; & \vdots & \\ r_{n1} & \cdots & r_{np} \end{array}\right],\;E=\left[\begin{array}{ccc} e_{11} & \cdots & e_{1p}\\ e_{21} & \cdots & e_{2p}\\ \; & \vdots & \\ e_{m1} & \cdots & e_{mp} \end{array}\right].$$

In contrast to the colour matrix of Equation (6), which is simply the sum of the two sets, we instead seek to construct a colour signal matrix that includes all n×m possible combinations of {r(x)} and {e(x)}. We use the construction idea from Ref. [37] but apply it in the logarithmic domain.

First, consider the kth row of *E*, which is a single shading vector $e_{k}\left(x\right)$ with $k\in \left\{1,\cdots m\right\}$, and construct an n×p matrix $E_{k}$ with *n* identical rows, each defined by the chosen $e_{k}\left(x\right)$. Its matrix elements can be written as

(12) $$E_{k}=\left[\begin{array}{ccc} e_{k1} & \cdots & e_{kp}\\ e_{k1} & \cdots & e_{kp}\\ \; & \vdots & \\ e_{k1} & \cdots & e_{kp} \end{array}\right].$$

Now, the total colour signal matrix *C* can be expressed as the sum of two large concatenated albedo and shading matrices $R_{c}$ and $E_{c}$,

(13) $$C=E_{c}+R_{c},$$

where

(14) $$E_{c}=\left[\begin{array}{c} E_{1}\\ E_{2}\\ \vdots \\ E_{m} \end{array}\right],\;R_{c}=\left[\begin{array}{c} R\\ R\\ \vdots \\ R \end{array}\right].$$

$E_{c}$ is the concatenation of *m* different shading matrices $E_{k}$ defined by Equation (12) with *k* = 1,⋯m, and $R_{c}$ is the concatenation of *m* identical albedo matrices *R* defined by Equation (11). Each matrix in Equation (13) has dimension (m×n)×p. This equation is a generalisation of Equation (6).

### 3.2. Least Squares Solution

We seek the p×p linear matrix operator $L_{r}$,

(15) $$CL_{r}\approx R_{c}.$$

This is analogous to Equation (7), where *C* is now the concatenated colour matrix of Equation (13) and $R_{c}$ replaces *R*. In this case, by over-constraining the system so that n×m≫p, the least squares solution is

(16) $$L_{r}={\left(C^{⊤}\;C\right)}^{\;-1}\;C^{⊤}\;R_{c}.$$

Again, a 1d convolution filter $f_{r}$ can be extracted by taking the central column of $L_{r}$.

(As an aside, it is also possible to introduce a matrix operator $L_{e}$ that best recovers the shadings,

(17) $$CL_{e}\approx E_{c},$$

which has least squares solution

(18) $$L_{e}={\left(C^{⊤}\;C\right)}^{\;-1}\;C^{⊤}\;E_{c},$$

from which a convolution filter $f_{e}$ can be extracted. Since we are in the logarithmic domain, utilising Equation (13) reveals that $L_{r}$ + $L_{e}$ = *I*, where *I* is the p×p identity matrix. Consequently, the centre/surround convolution filters $f_{r}$ and $f_{e}$ sum to give a delta function,

(19) $$f_{r}+f_{e}=\delta \left(x-x_{0}\right),$$

where $x_{0}$ is the filter centre. This differs from typical centre/surround formulations where the radial surround is chosen to integrate to unity [6]).

Clearly from Equation (16), the least squares solution is seen to fundamentally depend upon the colour signal autocorrelation matrix $C^{⊤}\;C$ and the cross-correlation matrix $C^{⊤}\;R_{c}$. Physically, each matrix element ${\left[C^{⊤}\;C\right]}_{ij}$ describes how colour signal values are correlated at pixel locations i, j in the image training set, while each ${\left[C^{⊤}\;R_{c}\right]}_{ij}$ analogously describes how colour signal values are correlated with albedo values.

As shown next, in order to obtain a closed-form solution for $L_{r}$, we must perform a decomposition of $C^{⊤}\;C_{c}$ and $C^{⊤}\;R_{c}$ into terms that can themselves be evaluated in closed form.

### 3.3. Analytic Decomposition

Using Equation (13), the colour signal autocorrelation matrix is seen to be related to the cross-correlation terms in the following way:

(20) $$C^{⊤}\;C=C^{⊤}\;E_{c}+C^{⊤}\;R_{c}.$$

Crucially, it is shown in Appendix A that the cross-correlation terms can be decomposed as follows:

(21) $$\begin{array}{cc} C^{⊤}\;E_{c} & =E^{⊤}\;E+{\langle R\rangle }^{\;\;⊤}\;\;\langle E\rangle \\ C^{⊤}\;R_{c} & =\;R^{⊤}\;R\;+{\langle E\rangle }^{\;\;⊤}\;\;\langle R\rangle . \end{array}$$

- $C^{⊤}\;C$ is the colour signal autocorrelation matrix for the set of all m×n colour signals,
- $E^{⊤}\;E$ is the shading autocorrelation matrix for the starting set of *m* vectors {e} defined by Equation (11),
- $R^{⊤}\;R$ is the albedo autocorrelation matrix for the starting set of *n* vectors {r} defined by Equation (11),
- 〈E〉 is a row vector defined by the mean of the set {e},
- 〈R〉 is a row vector defined by the mean of the set {r}.

Now, substituting Equations (20) and (21) into Equation (16) yields the following expression for the least squares matrix operator $L_{r}$:

(22) $$L_{r}={\left(E^{⊤}\;E+{\langle R\rangle }^{\;\;⊤}\;\;\langle E\rangle +R^{⊤}\;R+{\langle E\rangle }^{\;\;⊤}\;\;\langle R\rangle \right)}^{\;\;-1}\left(R^{⊤}\;R\;+{\langle E\rangle }^{\;\;⊤}\;\;\langle R\rangle \right).$$

Since the mean terms 〈R〉 and 〈E〉 can be straightforwardly evaluated, the practical utility of this equation is the resulting separation between the shading and albedo information. As shown next (Section 3.4 and Section 3.5), the given functional forms for the possible albedo and shading training vectors, closed-form expressions for $E^{⊤}\;E$ and $R^{⊤}\;R$ can be derived by letting the number of training vectors m, n→∞ and analytically integrating over the entire parameter space. In other words, the training set will include *all* possible instances of the training vectors.

### 3.4. Shading Autocorrelation Matrix

Given a functional form for the shading training vectors $\left\{e^{\prime }\left(x\right)\right\}$, the shading autocorrelation matrix elements in the logarithmic domain can, in principle, be evaluated by integrating as follows:

(23) $${\left[E^{⊤}\;E\right]}_{ij}=\int_{u}^{v}p(e^{\prime })\;e_{i}\;e_{j}\;de^{\prime },$$

where $e_{i}$ = $\log(e_{i}^{\prime })$ and $e_{j}$ = $\log(e_{j}^{\prime })$ are the (logarithmic) values of the shading vectors at pixels *i* and *j* and $p(e^{\prime })$ is the probability density function for shadings taking values in the range [u,v], where u>0 and v>u, e.g., [u,v] = (0,1].

However, in order to derive a simple closed-form solution, a simpler way to proceed is to assume that $e_{i}\approx e_{i}^{\prime }$ and to use logarithmic units so that the interval [u,v] is replaced by $\left[\log u,\log v\right]$. Now we can replace the above equation with the following:

(24) $${\left[E^{⊤}\;E\right]}_{ij}\approx \int_{\log u}^{\log v}p(e^{\prime })\;e_{i}^{\prime }\;e_{j}^{\prime }\;de^{\prime }.$$

A suitable way to model training vectors (scan lines) through shadings that might be encountered in the real world without abrupt changes is to use slowly varying sinusoids. Consider training vectors of length *p* pixels defined by the following function:

(25) $$e_{i}^{\prime }=\frac{A}{2}+\frac{A}{2}\sin\left(kx+\phi \right),$$

where *x* is a positional coordinate that can be expressed in terms of pixels {i} along a 1d scan line (in any direction, as illustrated in Figure 3),

(26) $$x=\frac{i-1}{p-1},\;i=1,2,\cdots p.$$

**Figure 5:**
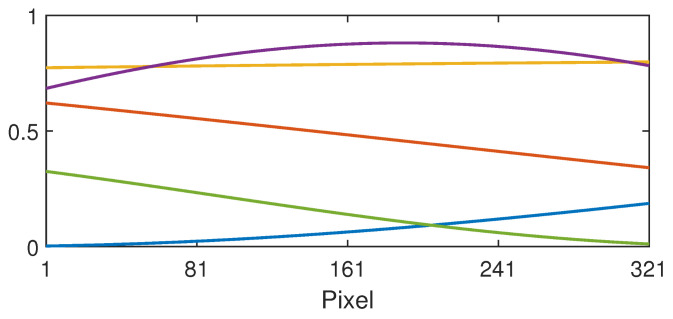
Example sinusoidal shadings (denoted by curves of different colours) in the range [u,v] = [0,1], defined by Equation (25) with the minimum wavelength of $\lambda_{\min}$ = 2, i.e., twice the length of the training vectors (*p* = 321 pixels). Evidently, many of these sinusoids are approximately straight line gradients.

Here, *A* is the amplitude in the interval [logu,logv] and the wavenumber is defined by *k* = 2π/λ in the interval $\left[0,k_{\max}\right]$, where λ is the wavelength and ϕ is the phase. Here, the maximum wavenumber is defined by $k_{\max}$ = $1/\lambda_{\min}$, where $\lambda_{\min}$ is the minimum wavelength. For example, we could choose $\lambda_{\min}$ = 2, which would mean that sinusoids with a wavelength smaller than twice the length of the training vectors (*p* pixels) are excluded from the training set. The function defined by Equation (25) is bounded in the interval [logu,logv]. Examples are shown in Figure 5 using the corresponding non-log units (where it is bounded in the interval [u,v]).

The probability density function $p(e^{\prime })$ depends upon those for the amplitude *A* in the range [log *u*, log *v*], the phase ϕ, and the wavenumber *k*. Substituting Equation (25) into (24) leads to the following volume integral:

(27) $$\begin{array}{cc} {\left[E^{⊤}\;E\right]}_{ij}= & \;\frac{A^{2}}{4}\int_{\log u}^{\log v}\int_{0}^{2\pi }\int_{0}^{k_{\max}}p\left(A\right)\;p\left(\phi \right)\;p\left(k\right)\\ & \times \left(1+\sin\left(kx+\phi \right)\right)\left(1+\sin\left(ky+\phi \right)\right)\;dA\;d\phi \;dk, \end{array}$$

where *x* depends upon *i* according to Equation (26) and, similarly, *y* = (j−1)/(p−1) with *j* = 1,2,⋯p. For uniform probability distributions, we have

(28) $$p\left(A\right)=\frac{1}{\log v-\log u},\;p\left(\phi \right)=\frac{1}{2\pi },\;p\left(k\right)=\frac{1}{k_{\max}}.$$

By integrating, in turn, over the amplitude, phase (where several terms evaluate to zero), and wavenumber (utilising the identity sin(A)sin(B) = $\frac{1}{2}\cos\left(B-A\right)$ − $\frac{1}{2}\cos\left(B+A\right)$), we arrive at the final result:

(29) $${\left[E^{⊤}\;E\right]}_{ij}=\;\frac{\log^{2}u+\log u\log v+\log^{2}v}{12}\;\left(1+\frac{\sin\left(k_{\max}\left(y-x\right)\right)}{2\;k_{\max}\left(y-x\right)}\right).$$

The mean shading vector required by Equation (22) can be evaluated as

(30) $$\langle E\rangle =\;\frac{A}{2}\int_{\log u}^{\log v}\int_{0}^{2\pi }\int_{0}^{k_{\max}}p\left(A\right)\;p\left(\phi \right)\;p\left(k\right)\left(1+\sin\left(kx+\phi \right)\right)\;dA\;d\phi \;dk.$$

This turns out simply to be a constant (for all {i}), as defined by

(31) $$\langle E\rangle =\frac{\log u+\log v}{4}.$$

**Figure 6:**
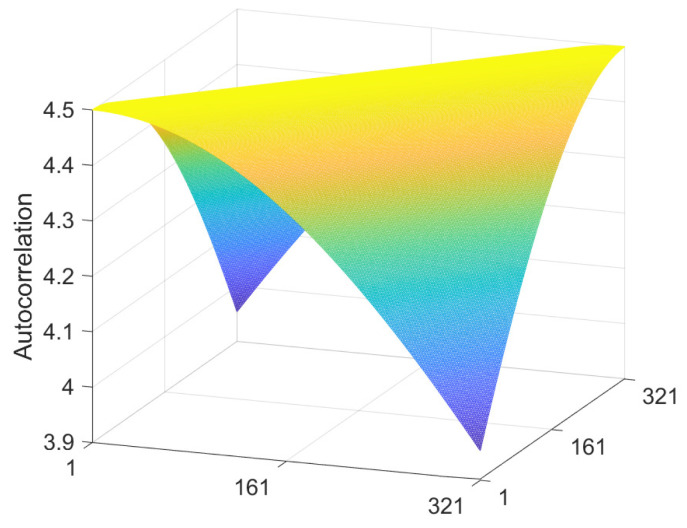
Shading autocorrelation matrix for sinusoids defined by Equation (25) with *p* = 321, $\lambda_{\min}$ = 2, and logarithmic units in the interval [−6, 0], which corresponds to [0.0025, 1] in non-log units.

The autocorrelation matrix $E^{⊤}\;E$, Equation (29), is illustrated as a mesh plot in Figure 6, where the minimum wavelength was taken to be $\lambda_{\min}$ = 2, i.e., twice the length of the training vectors. It has a Toeplitz structure due to the shift invariance that arises from the integration over phase ϕ. Clearly, the autocorrelation decreases with distance from the main diagonal due to the reduced correlation between pixel values separated by a sinusoidal function with a minimum wavelength of $\lambda_{\min}$ = 2.

For completeness, in Appendix B, the autocorrelation matrix for straight line gradients (linear ramps) is also derived. This matrix will not be shift-invariant since the ramps cannot be shifted by a phase within the bounds. It is possible to use shadings that are a weighted combination of sinusoids and linear ramps simply by weighting the autocorrelation matrices accordingly.

### 3.5. Albedo Autocorrelation Matrix

For a given image dataset, recall from the beginning of Section 2 that the colour signal autocorrelation matrix can be calculated numerically by using vectors that correspond to 1d scan lines of length *p* pixels taken from the images. By constructing the composite n×p vector *C*, where *N* is the number of data values (or scan lines) per component [38], it can be algebraically expressed as

(32) $$C^{\;⊤}\;C=\frac{1}{N}\;\left[\begin{array}{cccc} \sum_{k=1}^{N}c_{k1}^{2} & \sum_{k=1}^{N}c_{k1}\;c_{k2} & \cdots & \sum_{k=1}^{N}c_{k1}\;c_{kp}\\ \sum_{k=1}^{N}c_{k2}\;c_{k1} & \sum_{k=1}^{N}c_{k2}^{2} & \cdots & \sum_{k=1}^{N}c_{k2}\;c_{kp}\\ \vdots & \vdots & \ddots & \vdots \\ \sum_{k=1}^{N}c_{kp}\;c_{k1} & \sum_{k=1}^{N}c_{kp}\;c_{k2} & \cdots & \sum_{k=1}^{N}c_{kp}^{2} \end{array}\right]$$

In Ref. [39], it was found that for large image datasets such as ImageNet [40], shading gradients are typically minimal on average in the central region of the images where the autocorrelation matrix is found to have a Toeplitz structure. In other words, the *albedo* autocorrelation matrix $R^{\;⊤}\;R$ for large datasets might be assumed to be a Toeplitz matrix.

Recall that Hurlbert and Poggio used Mondrian images as the albedo images in their example training set [30]. Mondrian images consist of random arrangements of rectangular patches of various sizes [2] and have been widely used in visual experiments [41,42,43,44]. (Their appearance is inspired by the abstract grid-based paintings of the Dutch artist Piet Mondrian that first appeared in the early 1920s). Interestingly, it was found in Ref. [39] that, for a particular construction of Mondrian images, the autocorrelation matrix for Mondrian image datasets is a Toeplitz matrix. Furthermore, it is possible to find Mondrian datasets that have the same Toeplitz matrix as real image datasets. In other words, a statistical model for the autocorrelation matrix for Mondrian datasets, which can be obtained in closed form (as shown in Ref. [39]), can be used as a proxy for that of real image datasets.

Following Ref. [39], scan lines through Mondrian images can be modelled by introducing a correlation between adjacent pixels via a “step” parameter α, where 0≤α≤1. For a given pixel *i*, this describes the probability that the adjacent pixel takes on the same value, $p\left(r_{i+1}=r_{i}\right)$ = α. The probability that $r_{i+1}$ uniformly takes any other value in the range [a,b] instead is then 1−α. For general pixels i, j, it follows that

(33) $${\left[R^{⊤}\;R\right]}_{ij}=\int_{a}^{b}p(r^{\prime })r_{i}\;dr^{\prime }\left(\alpha^{\left|j-i\right|}r_{i}+\left(1-\alpha^{\left|j-i\right|}\right)\;\int_{a}^{b}p(r^{\prime })r_{j}\;dr^{\prime }\right),$$

where *r* = $\log(r^{\prime })$. Assuming a uniform probability distribution so that $p(r^{\prime })$ = 1/(b−a),

(34) $$\begin{array}{cc} {\left[R^{⊤}\;R\right]}_{ij}= & \;\frac{\alpha^{\left|j-i\right|}}{b-a}\left(b\left(\log^{2}b-2\log b+2\right)-a\left(\log^{2}a-2\log a+2\right)\right)\\ \; & +\frac{1-\alpha^{\left|j-i\right|}}{{\left(b-a\right)}^{2}}{\left(b\left(\log b-1\right)-a\left(\log a-1\right)\right)}^{2}. \end{array}$$

If [a,b] = (0,1], then

(35) $${\left[R^{⊤}\;R\right]}_{ij}=1+\alpha^{\left|j-i\right|}.$$

Physically, the step parameter α controls the average or expected size of the steps in the scan lines and therefore the expected size of the Mondrian patches. The expected step size *s* along a scan line is related to α as follows [39]:

(36) $$s=\frac{1}{1-\alpha }.$$

**Figure 7:**
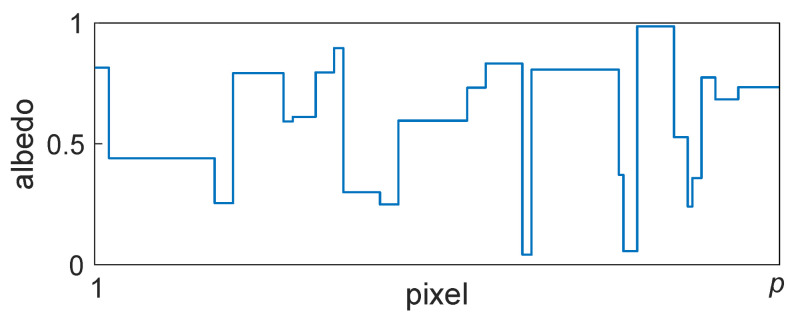
Example scan line of length *p* pixels through a Mondrian image with albedo values in the range [a,b] = (0,1].

**Figure 8:**
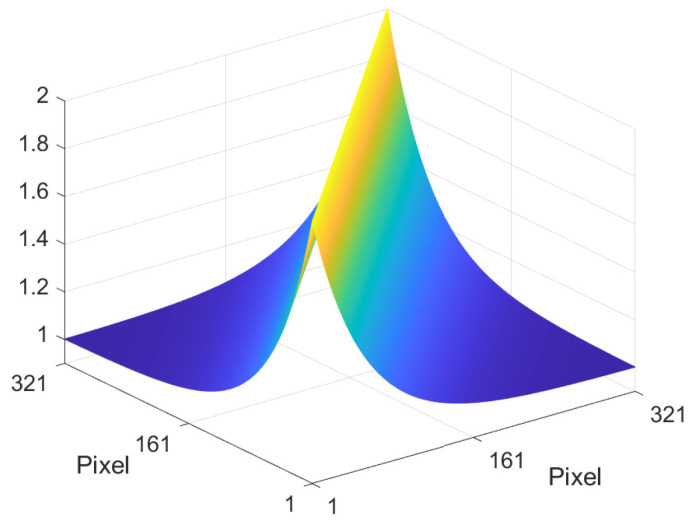
Albedo autocorrelation matrix in the logarithmic domain for Mondrians with α = 0.981, which corresponds to an expected step length of 52.6 pixels. The primal domain albedo values were restricted to the range [0,1].

An example scan line is illustrated in Figure 7. When α = 0, all pixel values are uncorrelated, and so *s* = 1. This corresponds to a completely random Mondrian (or random real scene). Accordingly, the autocorrelation matrix has maximum value along the main diagonal and minimum value elsewhere. When α is increased, the correlation between adjacent pixels increases and so the expected size of the Mondrian patches also increases. In other words, a larger α corresponds to real scenes that contain larger regions of constant albedo values on average. Figure 8 illustrates how the autocorrelation matrix decreases to its minimum value at a greater distance from the main diagonal for a larger α value. In Section 3.7, it is shown how this directly impacts the shape of the optimum filter.

Since the derivation of Equation (34) assumed a uniform probability distribution for the albedo values, the mean albedo vector required by Equation (22) is given by

(37) $$\langle R\rangle =\frac{1}{b-a}\int_{a}^{b}r_{i}\;dr^{\prime }\;=\frac{1}{b-a}\;\left(b\left(\log b-1\right)-a\left(\log a-1\right)\right),$$

which is a constant for all {i}. However, it is likely that the mean albedo of the image dataset differs from this value, in which case a scale and offset least squares fit to the autocorrelation matrix for the dataset can be performed [39]. For consistency, 〈R〉 would then need to be estimated using the image dataset instead of Equation (37). This procedure is discussed further in the next section, which describes implementation details for the method.

### 3.6. Implementation

#### 3.6.1. Designing a Filter

Given an image dataset for a specific category or type of scene, the goal of the algorithm described below is to design an optimum filter for that scene category, which could also be applied to other unseen images that fall under that category.

1. Linearise the input images by inverting the gamma encoding curve and calculate the luminance channel as the appropriate weighted sum of the RGB channels.
2. Based upon an estimate for the nature of the shadings present in the dataset, calculate the shading autocorrelation matrix $E^{⊤}\;E$ and mean vector 〈E〉, for example by using Equations (29) and (31). Considerations include the following:
  - The type of shadings present such as sinusoids, linear ramps, or a weighted combination. For sinusoids, the minimum wavelength can be changed.
  - The spatial extent of the shadings (in pixels). This corresponds to the length of the scan lines and hence the diameter of the output filter.
  - The shading value limits [logu,logv]. If the image pixel values have been normalised to the range [0,1] in the primal domain, then *v* can be taken to be 1 and an estimate can be made for *u* before converting to logarithmic units.
3. Calculate the albedo autocorrelation matrix $R^{⊤}\;R$ and mean vector 〈R〉. To do this,
  (a) First, calculate $C^{⊤}\;C$ and the mean vector 〈C〉 for the dataset (logarithm of the luminance channel) numerically using Equation (32). For every image in the dataset, the scan lines (training vectors) of length *p* can be extracted by rotating the images through all 360 single degree increments and taking a scan line from a fixed position each time, for example by choosing the horizontal line that passes through the centre of the images.
  (b) Estimate $R^{⊤}\;R$ by rearranging Equation (A4),
    (38) $$R^{⊤}\;R=C^{⊤}\;C-\left(E^{⊤}\;E+{\langle R\rangle }^{\;\;⊤}\;\;\langle E\rangle +{\langle E\rangle }^{\;\;⊤}\;\;\langle R\rangle \right),$$
    where 〈R〉 can be evaluated as
    (39) $$\langle R\rangle =\langle C\rangle -\langle E\rangle .$$
  (c) In order to obtain a perfectly smooth closed-form solution, find the closest Mondrian autocorrelation matrix. This can be achieved by applying scale and offset parameters to Equation (34) and then performing a least squares fit to Equation (38). The mean vector 〈R〉, which will be approximately constant, can be smoothed by averaging its elements if necessary.
4. Calculate the matrix operator $L_{r}$ using Equation (22). Use the central column as the 1d albedo filter and convert this to 2d.

Note that when calculating the matrix operator $L_{r}$, taking the pseudoinverse of $C^{⊤}\;C$ can lead to a discontinuity at the filter edges when $C^{⊤}\;C$ has steep transitions, for example when α is large. This is due to a natural consequence of the inverse of Toeplitz matrices [45]. The discontinuities can either be omitted, which has negligible effect on the overall effect of the filter, or be eliminated by introducing a regularisation term that favours continuity when solving the regression. In the latter case, Equation (16) is generalised to

(40) $$L_{r}=\left({\left(C^{⊤}\;C\right)}^{\;-1}+\gamma D^{⊤}\;D\right)\;C^{⊤}\;R_{c},$$

where *D* is the derivative matrix operator and γ is the minimum scalar that eliminates the discontinuity.

#### 3.6.2. Filtering an Image

In order to filter an input image in practice,

1. Linearise by inverting the encoding gamma curve and calculate the luminance channel *Y* as the appropriate weighting of the RGB channels. Take the logarithm of the luminance channel.
2. Transform the log luminance image to the Fourier domain and multiply by the Fourier transform of the 2d filter (zero padded if necessary) before converting back to the primal domain. Since artefacts can arise from discontinuities at the image boundaries due to the non-periodic nature of a typical real-world image, a computational trick to remove these is to first convert the image into a continuous image that is four times as large by mirroring in the horizontal and vertical directions [33].
3. Subtract the 99.7th quantile in order that the maximum value of the log luminance image be zero. (Any values larger than zero should be clipped to zero). This generally produces a lighter image, which is useful from an image preference point of view.
4. Exponentiate the filtered log luminance image from the previous step. Use the original RGB channels (colour signals) together with the filtered luminance channel to calculate a filtered colour image, appropriately scaling for the new luminance. Mathematically,
  (41) $${\hat{c}}_{i}\left(x,y\right)=c_{i}\left(x,y\right)\times \frac{\hat{Y}\left(x,y\right)}{Y(x,y)},$$
  where $\left\{{\hat{c}}_{i}\right\}$ with *i* = R, G, or B are the output colour signals, $\left\{c_{i}\right\}$ with *i* = R, G, or B are the corresponding input colour signals, *Y* is the input luminance channel, and $\hat{Y}$ is the filtered output luminance channel. This colour mapping preserves chromaticity [11]. Finally, reapply the gamma encoding curve and renormalise the image to the desired range as required.

### 3.7. Filter Shape

In this section, we have shown that Hurlbert and Poggio’s least squares approach to determining an optimum filter for removing illumination gradients or shading from images can be reformulated so that the optimisation can be solved in closed form. In particular, the optimisation was seen to directly depend upon the autocorrelation statistics of the albedo and shading components of the images in the training set.

An example model for the shading autocorrelation matrix $E^{⊤}\;E$ was derived, where the training vectors (scan lines) were taken to be sinusoids or linear ramps. Significantly, the closed-form solution was obtained by integrating over all possible training vectors. In other words, an infinitely large training set was utilised.

By making an analogy between real image datasets and Mondrian image datasets, a model for the albedo autocorrelation matrix $R^{⊤}\;R$ was derived where the α parameter controls the average size of the Mondrian patches or equivalently models the average size of constant regions in real images. Again, the closed-form solution was obtained by integrating over all possible training vectors.

**Figure 9:**
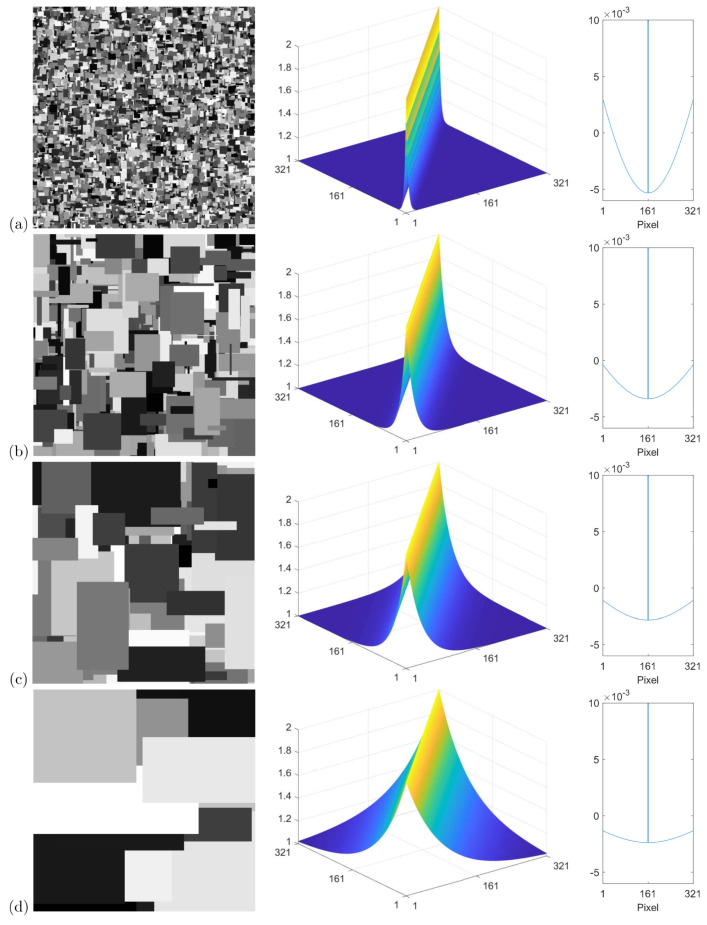
For the selection of α values given in the main text, each row (denoted by (**a**), (**b**), (**c**), or (**d**)) shows an example Mondrian albedo image (**left**), the corresponding albedo autocorrelation matrix (**centre**), and the optimised filter (**right**). Here, a p×p pixel grid was used with *p* = 321.

An important finding is that the shapes of both $E^{⊤}\;E$ and $R^{⊤}\;R$ directly impact the shape of the optimised filter. To illustrate this, consider a fixed $E^{⊤}\;E$ for a 50:50 mix of sinusoids and linear ramps, with an illumination range of [u,v] = [0.0025,1]. Now, consider $R^{⊤}\;R$ for a selection of different α values with albedo range [a,b] = (0,1]. Row (a) of Figure 9 shows an example Mondrian image for α = 0.788 (left figure), which corresponds to an expected step length of *s* = 4.7 pixels according to Equation (36), along with $R^{⊤}\;R$ (centre figure) and a cross-section of the 1d filter obtained from the optimisation (right figure). Clearly, $R^{⊤}\;R$ is narrow and the filter surround is deep in order to capture the relatively local changes in albedo. In row (b), α = 0.942, which corresponds to *s* = 17.2 pixels. Evidently, $R^{⊤}\;R$ widens and the filter becomes shallower as changes in albedo become less localised on average. This trend continues for row (c), where α = 0.942 and *s* = 34.5 pixels, and row (d), where α = 0.988 and *s* = 83.3 pixels.

## 4. Results and Discussion

In this section, we present an experimental evaluation of our method. Firstly, in order to evaluate the performance of the method objectively, we perform an experiment where we use pages of text extracted from journal articles and books in PDF format. Since these do not contain any shading, they can be used as a synthetic albedo ground truth. By superimposing randomly generated synthetic shadings with a known functional form on these pages and determining the optimised filter from the autocorrelation matrices, the ability of the method to remove the shadings can be quantified.

Secondly, the qualitative ability of the method to mitigate shadings is investigated for a challenging real-world image dataset (TM-DIED) by following the implementation procedure described in Section 3.6.

### 4.1. Text Image Processing

**Figure 10:**
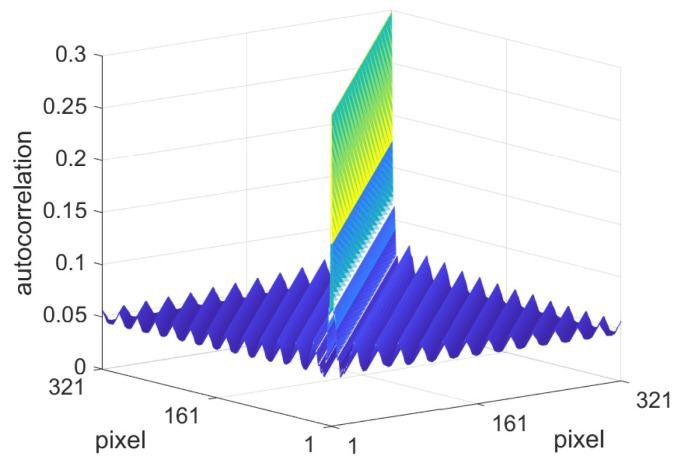
Autocorrelation matrix $R^{⊤}\;R$ (in the logarithmic domain) for the dataset of text images on a p×p pixel grid, where *p* = 321.

One might anticipate that an autocorrelation matrix for pages of text will very quickly decrease to its minimum value away from the matrix diagonal; in other words, the peak along the diagonal will be very narrow due to the fact that the lines of white space between lines of text are short-ranged on average. This is indeed seen to be the case in Figure 10, which shows the synthetic $R^{⊤}\;R$ trained on 50 randomly selected pages from our dataset comprised of 3500 pages from randomly selected journal articles and books. The pages were extracted at a resolution such that the page width was 641 pixels and the data were normalised to the range [a,b] = (0,1] before taking the logarithm. Note that since we are determining a convolution filter, our dataset should be approximately shift-invariant on average. In order to remove any overall order that could arise from page borders and column spaces, the scan lines were extracted from a central p×p crop, where *p* = 321 pixels, i.e., half the page width. This enabled shifts of the 50 sampled pages to be included in the autocorrelation matrix calculation, i.e., 160 shifts to the left and 160 to the right, which includes the corresponding vertical shifts from the included rotations.

Along with the very narrow peak along the diagonal, observe that the autocorrelation does not directly fall to its minimum value, instead decaying in a manner resembling a wave. This is due to the periodicity of the lines of text, which will remain even when shifts are included. Since the frequency of the lines of text is not the same in each page, this wave structure represents the average frequency of the lines of text in the 50 sampled pages from the dataset.

**Figure 11:**
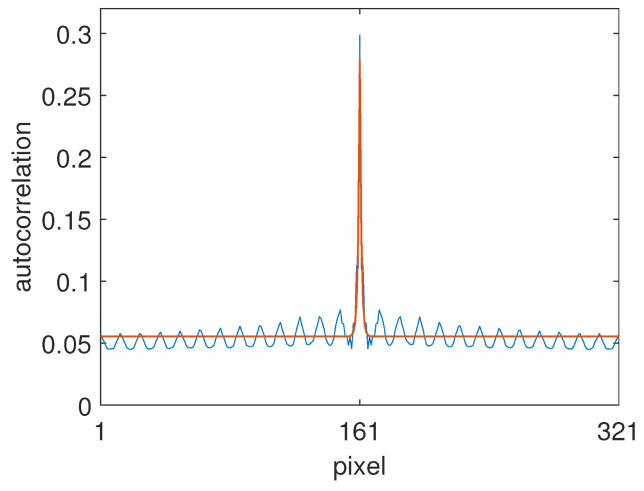
Diagonal cross section of the numerically determined $R^{⊤}\;R$ (blue line) along with the best Mondrian fit (red line).

In order to obtain a closed-form expression for $R^{⊤}\;R$, we applied scale and offset parameters to Equation (34) and then used least squares to find the Mondrian autocorrelation matrix that is the closest representation of our numerically determined text autocorrelation matrix. Cross sections of the diagonals of the two matrices are shown in Figure 11. Evidently, the Mondrian model is able to provide a good fit for the narrow peak with α = 0.594, which corresponds to an average step of 2.5 pixels according to Equation (36). The Mondrian model is unable to capture the wave structure mentioned above, but, in any case, we would expect the oscillations to eventually disappear if the size of the training set were to be increased. The value of 〈R〉 was determined numerically to be −0.1557 for all {i}.

Slowly varying sinusoids were used for the shadings (with the minimum wavelength taken to be 4p, i.e., four times the length of the scan lines or training vectors), and so $E^{⊤}\;E$ and 〈E〉 are given by Equations (29) and (31).

**Figure 12:**
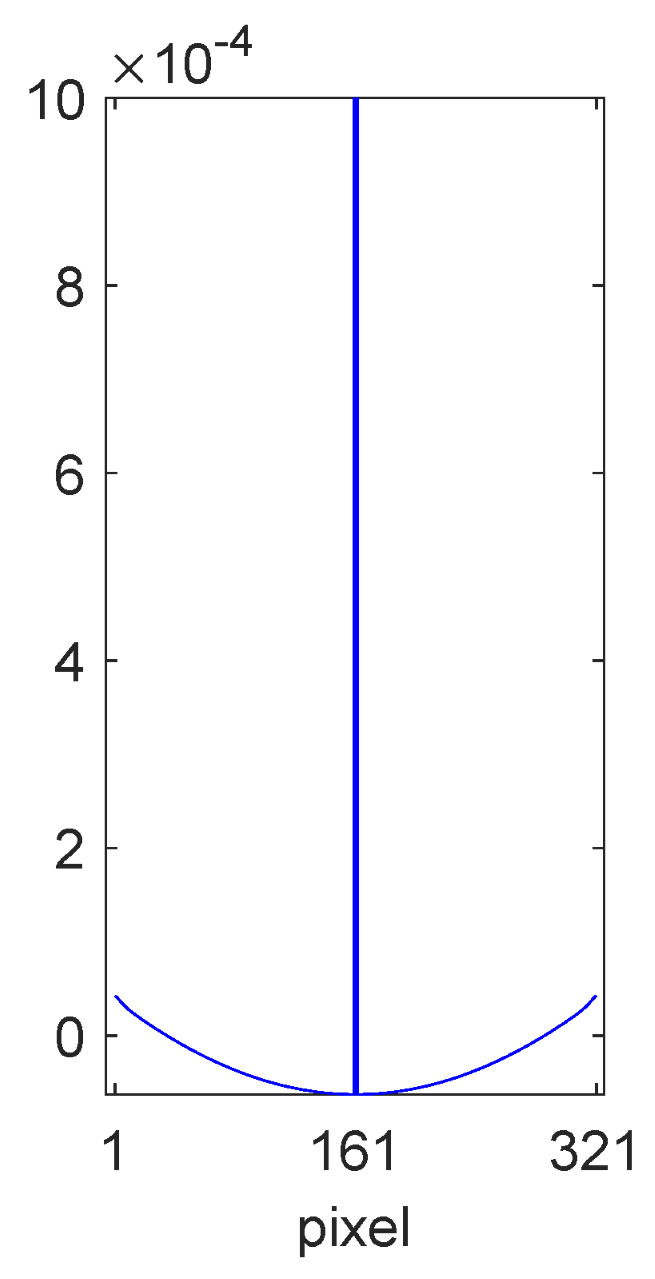
Cross section of the optimised 2d filter, $f_{2d}$, for the text image dataset.

**Figure 13:**
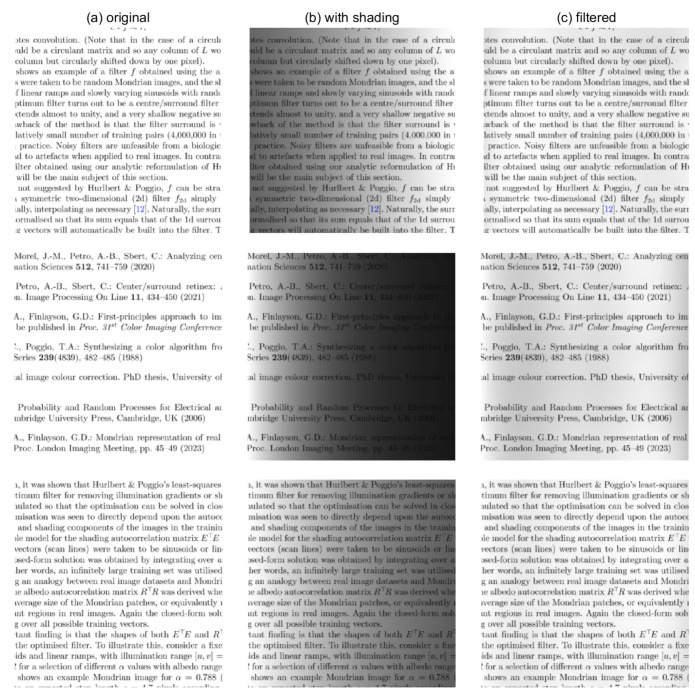
Three example results using a draft copy of this manuscript. (**a**) Original PDF pages. (**b**) Colour signals (i.e., with randomly generated shading superimposed). (**c**) Filtered results.

Figure 12 illustrates a cross section of the optimised filter $f_{2d}$ for our text dataset, which was determined using Equation (22) before converting to 2d. In order to visualise the type of performance to be expected, randomly generated shadings were superimposed on pages from a draft PDF copy of the present manuscript. Several results of filtering these pages are illustrated in Figure 13. It can be seen that the filter does well at removing illumination gradients and in general reproduces text without visible artefacts. However, white areas are generally reproduced darker than they appear in the ground truth. We would expect improved performance for a filter optimised for the manuscript itself.

To quantify the performance, let us denote the *i*th colour signal by $C_{i}^{\prime }$, which is the product of the *i*th sinusoidal shading image $E_{i}^{\prime }$ (randomly generated) and $R_{i}^{\prime }$, the latter being the luminance channel of the *i*th text image (synthetic albedo) from the dataset. Note that here we have reintroduced the prime symbols to indicate that the logarithm has not yet been taken. Let ${\hat{R}}_{i}$ denote the albedo image estimated by convolving $C_{i}$ = $\log C_{i}^{\prime }$ with the optimised filter $f_{2d}$, then

(42) $${\hat{R}}_{i}=\exp\left(C_{i}\;★\;f_{2d}\right).$$

We would like to measure how close ${\hat{R}}_{i}$ is to $R_{i}$. It should be noted that after performing the convolution, we can arrive at the same colour image $C_{i}$ given the pairs $\left(R_{i},E_{i}\right)$ and $\left(\alpha R_{i},\left(1-\alpha \right)E_{i}\right)$ as there is an in-built scaling ambiguity. Thus, in considering how close ${\hat{R}}_{i}$ is to $R_{i}$, let us allow a constant scaling term $k_{R,i}$ so that $∥k_{R,i}\;{\hat{R}}_{i}-R_{i}∥$ is minimised in a least squares sense. Here and in the next two equations, ∥.∥ denotes the Frobenius norm. Our percentage recovery error denoted by errorR is defined as

(43) $$errorR\left({\hat{R}}_{i},R_{i}\right)=100\times \frac{∥k_{R,i}\;{\hat{R}}_{i}-R_{i}∥}{∥R_{i}∥}.$$

Of course, we must compare the error in our method to the error found when the image $C_{i}$ is not filtered at all, i.e., when no action has been taken to remove shading. For consistency, we also allow a per image scaling term $k_{C,i}$ that is designed to minimise $∥k_{C,i}\;C_{i}-R_{i}∥$ in a least squares sense. Thus, the *null* error denoted by errorN is calculated as

(44) $$errorN\left(C_{i},R_{i}\right)=100\times \frac{∥k_{C,i}\;C_{i}-R_{i}∥}{∥R_{i}∥}.$$

**Figure 14:**
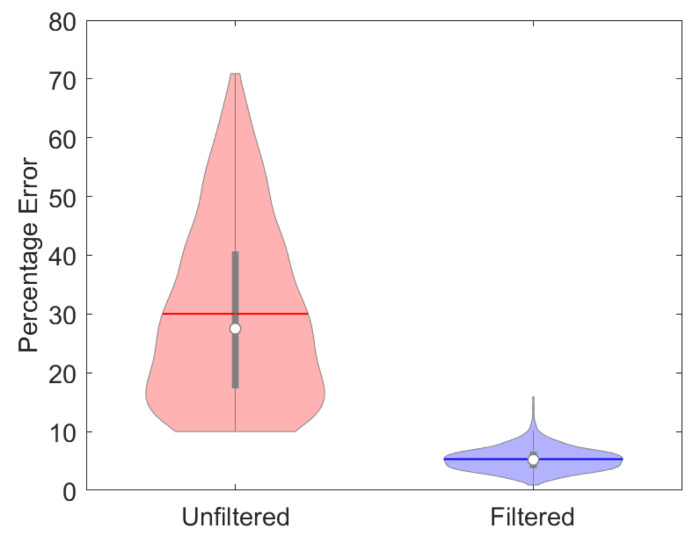
Violin plot showing the percentage error with and without the application of the filter.

For 1000 randomly selected images $\left\{C_{i}\right\}$, *i* = 1,2,⋯1000 (each with randomly generated shadings), the percentage recovery and null errors can be visualised in the violin plot of Figure 14. Note that only shadings with a mean null error above 10% were considered since below this threshold, the visual effect of shading was often not significant, but at 10%, the shading effect was always clearly evident. The mean of the null error (the error without filtering) on the LHS (pink violin) is seen to be 30%, whereas the mean of the percentage recovery error on the RHS (blue violin) is 5.31%, and so the application of the filter has reduced the overall error by a factor of over 5. Furthermore, the largest errors after filtering are much diminished, as indicated by the top section of the violins.

Although our method delivers good performance in terms of shading removal, as a simple linear convolution based on least squares optimisation, it cannot be expected to perform as well as a CNN-based method trained for this task [46].

### 4.2. Lightness Processing

Here, we test the ability of the method to mitigate shadings from a real-world image dataset, namely the TM-DIED dataset [32], which was designed to contain images taken in challenging lighting conditions.

**Figure 15:**
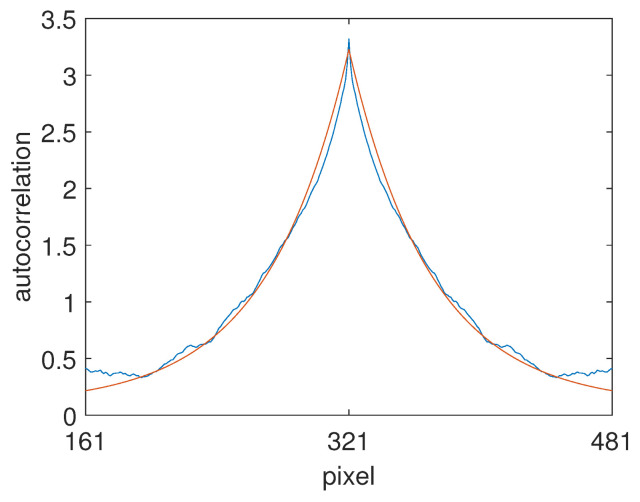
Diagonal cross-section of the fit between the numerically estimated albedo autocorrelation matrix (blue curve) and the Mondrian model with α = 0.99 (red curve).

Following the algorithm detailed in Section 3.6, we calculated the colour signal autocorrelation matrix $C^{⊤}\;C$ using scan lines from the 222 images in the dataset. (For convenience, the images were first resampled to 641 pixels on the shorter side). For the unknown shadings present, we assumed a 50:50 mix of slowly varying sinusoids and linear ramps in the range [logu,logv] = [−6,0], also 641 pixels in length. This provided an approximately smooth shift-variant estimate of $R^{⊤}\;R$ using Equation (38), which was then mapped to the closest-fitting analytic Mondrian autocorrelation matrix using the central quadrant. A diagonal cross-section of the fit is shown in Figure 15. The value for α was found to be α = 0.99. A cross-section of the resulting 641 by 641 pixel filter, $f_{2d}$, obtained from solving the optimisation, was shown in Figure 1.

**Figure 16:**
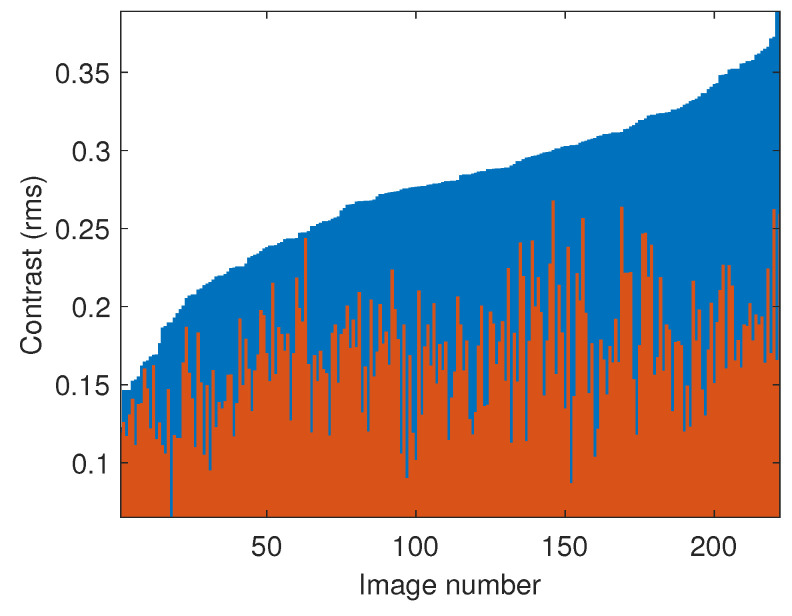
(blue bars) rms contrast of TM-DIED dataset images, arranged in order from low to high. (red bars) rms contrast of the corresponding filtered images.

Although there is no shading-free ground truth for the TM-DIED dataset, we would expect the removal of shadings to compress the dynamic range of the dataset images. Indeed, the dynamic range compression problem exists because of illumination. The dynamic range of reflectances is no more than 100 to 1. Yet, real scene luminance ratios can easily be 10,000 to 1 or higher. Input images with strong sunlight and deep shadows often lack detail when the images are rendered due to the limited dynamic range of the display. When we filter the images to remove shading (illumination gradients), we can see detail in the shadow and highlight regions. Intuitively, the standard deviation of the luma in the output images will be less than in the input. Indeed, the standard deviation of the luma channel, which is also known as the root mean square (rms) contrast [47], is an appropriate way to quantify dynamic range compression as it is a statistical measure that is not affected by outliers. Mathematically, it is defined as follows:

(45) $$C_{\mathrm{rms}}=\sqrt{\frac{1}{N}\sum_{k}^{N}{\left(Y_{k}^{\prime }-\bar{Y^{\prime }}\right)}^{2}},$$

where *k* = 1,⋯N denotes the *k*th pixel for image pixels arranged as a vector, $Y_{k}^{\prime }$ denotes the luma of the *k*th pixel normalised to the range [0,1], and ${\bar{Y}}^{\prime }$ is the average luma for all pixels in the image. Figure 16 shows a bar chart for the rms contrast calculated for each image in the dataset, both with and without application of the convolution filter, $f_{2d}$. The input images have been sorted in order of increasing rms contrast. It can be seen that the application of the filter does indeed reduce the rms contrast in all cases. The average rms contrast (i.e., the standard deviation of the luma channel averaged over all 222 images in the dataset) is reduced from 0.2759 to 0.1691.

**Figure 17:**
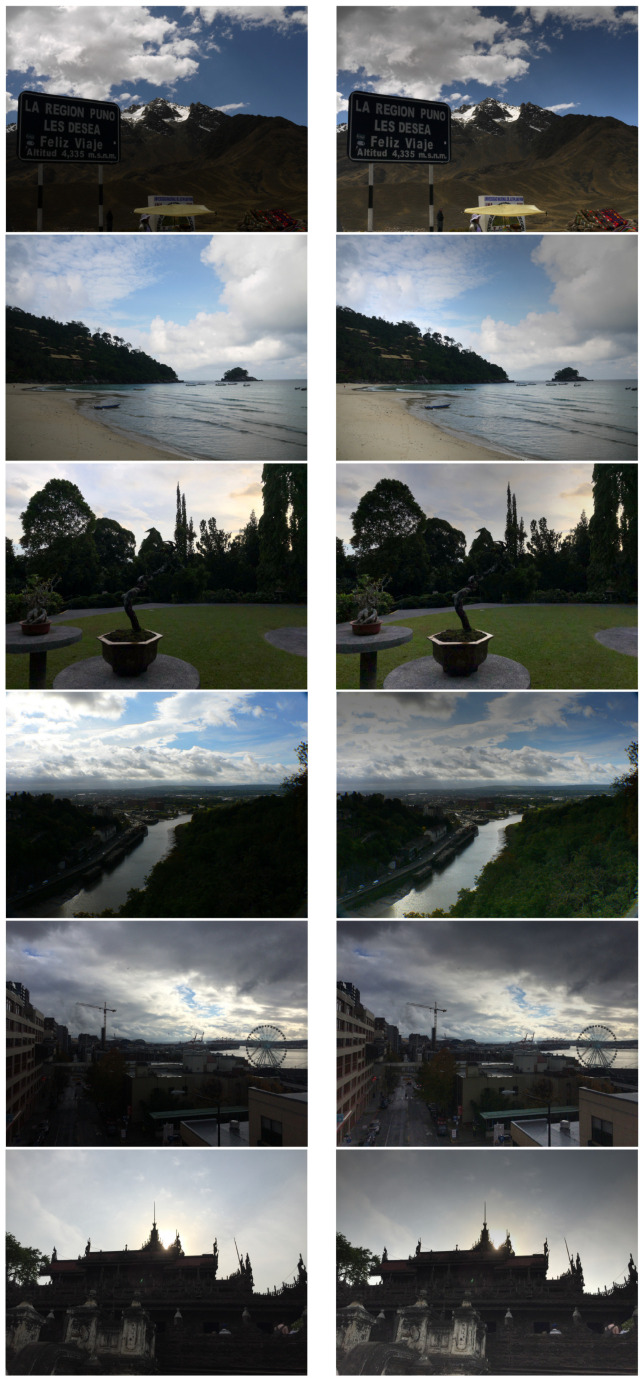
(**left**) Example images from the TM-DIED dataset [32]. (**right**) Same images processed using the filter of Figure 1.

Qualitative example results of applying the filter to images from the dataset are shown in Figure 17. It can be seen that the filter has subtly removed shading from the images without introducing obvious artefacts. Indeed, as stressed in the introduction, the original aim of retinex (as opposed to subjective image enhancement methods) was simply to mitigate gradients in the illumination.

## 5. Conclusions

In 1988, Hurlbert and Poggio [30] proposed a simple numerical method for finding an optimal linear filter that removes shading from images for a set of training examples. In this paper, we reformulated and further developed their approach by finding solutions in closed form, which has the dual advantages of effectively accounting for unseen data and in deriving smooth, as opposed to jagged, filters.

As one application, we designed a filter optimised for removing shading from text documents and used this to carry out an error analysis. We also designed a filter optimised for an image dataset produced in challenging lighting conditions and found that it could subtly remove shading. As future work, we intend to carry out further investigations into the lightness rendition afforded by the method.

Finally, we point out that although any variant of convolutional retinex is unlikely to deliver shading-free images or, indeed, preferred images, we point out that spatially varying tone-mapping algorithms, including edge-sensitive variants such as those that use bilateral filtering [48], make an assumption about how spatial information is integrated. Thus, our method could also be applied as a processing stage of more advanced algorithms.

## Data Availability

The dataset used in this article is not readily available because copyrighted text documents were used. However, any set of text documents can be used to generate a filter. The numerical values of the particular filter used in Figure 10 and Figure 11 can be obtained from the authors upon request.

## Appendix A. Analytic Decomposition

In order to derive Equation (21), it is useful to begin by explicitly writing out the matrix elements of the (m×n)×p colour signal matrix *C* defined by Equations (13) and (14):

(A1) $$C=\left[\begin{array}{c} \left[\begin{array}{cccc} E_{11}+R_{11} & E_{12}+R_{12} & \cdots & E_{1p}+R_{1p}\\ E_{11}+R_{21} & E_{12}+R_{22} & \cdots & E_{1p}+R_{2p}\\ \; & & \vdots & \\ E_{11}+R_{n1} & E_{12}+R_{n2} & \cdots & E_{1p}+R_{np} \end{array}\right]\\ \left[\begin{array}{cccc} E_{21}+R_{11} & E_{22}+R_{12} & \cdots & E_{2p}+R_{1p}\\ E_{21}+R_{21} & E_{22}+R_{22} & \cdots & E_{2p}+R_{2p}\\ \; & & \vdots & \\ E_{21}+R_{n1} & E_{22}+R_{n2} & \cdots & E_{2p}+R_{np} \end{array}\right]\\ \vdots \\ \left[\begin{array}{cccc} E_{m1}\;+\;R_{11} & E_{m2}\;+\;R_{12} & \cdots & E_{mp}\;+\;R_{1p}\\ E_{m1}\;+\;R_{21} & E_{m2}\;+\;R_{22} & \cdots & E_{mp}\;+\;R_{2p}\\ \; & & \vdots & \\ E_{m1}\;+\;R_{n1} & E_{m2}\;+\;R_{n2} & \cdots & E_{mp}\;+\;R_{np} \end{array}\right] \end{array}\right].$$

Here, submatrix brackets have been included for clarity. The second index in the subscripts denotes the positional coordinate, 1≤i≤p, where *p* is the length of the training vectors in pixels.

Since $C^{⊤}\;C$ is a p×p matrix, it follows that a general matrix element is given by multiplying the ith row of $C^{\;⊤}$ (where ⊤ denotes the transpose) by the jth column of *C*,

(A2) $$\begin{array}{cc} nm\;{\left[C^{⊤}\;C\right]}_{ij} & =\left(E_{1i}+R_{1i}\right)\left(E_{1j}+R_{1j}\right)\\ \; & \;+\left(E_{1i}+R_{2i}\right)\left(E_{1j}+R_{2j}\right)+\cdots \\ \; & \;+\left(E_{1i}+R_{ni}\right)\left(E_{1j}+R_{nj}\right)\\ \; & \;+\left(E_{2i}+R_{1i}\right)\left(E_{2j}+R_{1j}\right)\\ \; & \;+\left(E_{2i}+R_{2i}\right)\left(E_{2j}+R_{2j}\right)+\cdots \\ \; & \;+\left(E_{2i}+R_{ni}\right)\left(E_{2j}+R_{nj}\right)\\ \; & \;\vdots \\ \; & \;+\left(E_{mi}\;+R_{1i}\right)\left(E_{mj}\;+R_{1j}\right)\\ \; & \;+\left(E_{mi}\;+R_{2i}\right)\left(E_{mj}\;+R_{2j}\right)+\cdots \\ \; & \;+\left(E_{mi}\;+R_{ni}\right)\left(E_{mj}\;+R_{nj}\right). \end{array}$$

Here, autocorrelation has been defined to include a normalisation by the number of sample points, n×m. Collecting terms yields

(A3) $${\left[C^{⊤}\;C\right]}_{ij}=\frac{1}{m}\sum_{k=1}^{m}E_{ki}E_{kj}+\frac{1}{nm}\sum_{k=1}^{n}R_{ki}\sum_{k=1}^{m}E_{kj}+\frac{1}{n}\sum_{k=1}^{n}R_{ki}R_{kj}+\frac{1}{nm}\sum_{k=1}^{m}E_{ki}\sum_{k=1}^{n}R_{kj}.$$

This can be expressed in matrix form as follows:

(A4) $$C^{⊤}\;C=E^{⊤}\;E+{\langle R\rangle }^{\;\;⊤}\;\;\langle E\rangle +R^{⊤}\;R+{\langle E\rangle }^{\;\;⊤}\;\;\langle R\rangle .$$

Here, the angled brackets denote the mean value of each column of the matrix, and so $\langle E\rangle$ and $\langle R\rangle$ are row vectors of length *p* pixels.

A similar analysis as above shows that the first two terms of Equation (A4) can be identified as the colour signal and albedo cross-correlation term $C^{⊤}\;R_{c}$ of Equation (20), while the third and final term can be identified as the colour signal and shading cross-correlation term $C^{⊤}\;E_{c}$. This yields the decomposition given in Equation (21).

## Appendix B. Linear Ramps

Consider training vectors of length *p* pixels defined by the following function:

(A5) $$e_{i}^{\prime }=mx+c,$$

where *x* is a positional coordinate that can be expressed in terms of pixels {i} along a 1d scan line (in any direction) according to Equation (26). Here, *m* is the line gradient (which can be positive or negative), *c* is the offset, and only function values in the range [logu,logv] are permitted.

The probability density function $p(e^{\prime })$ depends upon those for *m* and *c*. Substituting Equation (A5) into (24) leads to the following surface integral:

(A6) $$\begin{array}{cc} {\left[E^{⊤}\;E\right]}_{ij}= & \int_{0}^{\log v-\log u}\;\int_{\log u}^{\log v-m}p\left(m\right)\;p_{1}\left(c\right)\;\left(mx+c\right)\left(my+c\right)dm\;dc\\ \; & +\int_{\log u-\log v}^{0}\;\int_{\log u-m}^{\log v}p\left(m\right)\;p_{2}\left(c\right)\;\left(mx+c\right)\left(my+c\right)dm\;dc, \end{array}$$

where *x* is related to pixel *i* via Equation (26), and, similarly, *y* = (j−1)/(p−1) with *j* = 1,2,⋯p. Here, the first term is for positive and the second is for negative gradients (in the range [u,v] before converting to log units). For uniform probability distributions, we have

(A7) $$\begin{array}{cc} p_{1}\left(c\right) & ={\left(\left(\log v-m\right)-\log u\right)}^{-1},\\ p_{2}\left(c\right) & ={\left(\log v-\left(\log u-m\right)\right)}^{-1},\\ p(m) & ={\left(2\left(\log v-\log u\right)\right)}^{-1}. \end{array}\;$$

By first integrating over the offset *c* and then over the gradient *m*, we arrive at

(A8) $${\left[E^{⊤}\;E\right]}_{ij}=\frac{\log u^{2}+\log u\log v+\log v^{2}}{3}\;+\frac{1}{3}\;\left(xy-\frac{\left(x+y\right)}{2}+\frac{1}{12}\right)\;{\left(\log v-\log u\right)}^{2}.$$

The mean shading vector required by Equation (22) is found by setting my+c = 1 in Equation (A6) and integrating, which yields the following constant for all {i}:

(A9) $$\langle E\rangle =\frac{\log u+\log v}{2}.$$
